# Complete resolution of recurrent piriformis syndrome after piriformis resection with 3 years’ follow up: A case report

**DOI:** 10.1016/j.ijscr.2020.11.099

**Published:** 2020-11-20

**Authors:** Achmad Fahmi, Mustaqim Apriyansa Rahmadhan, Dirga Rachmad Aprianto, Heri Subianto, Agus Turchan

**Affiliations:** a Faculty of Medicine, Universitas Airlangga, Surabaya, Indonesia; b Department of Neurosurgery, Dr. Soetomo General Academic Hospital, Surabaya, Indonesia; c Department of Surgery, Faculty of Medicine, Universitas Islam Sutan Agung, Semarang, Indonesia

**Keywords:** Piriformis syndrome, Piriformis resection, Pain relief

## Abstract

**Introduction:**

Piriformis syndrome (PS) is an uncommon neuromuscular condition characterized by buttock pain radiating to the leg. Although the goal of PS treatment is symptom relief, it is difficult in some patients using conservative treatment alone.

**Presentation of case:**

A 72-year-old male underwent piriformis resection after failed sequential steroid injection of the piriformis. The patient ultimately underwent surgery for resection of the piriformis muscle and experienced satisfactory pain relief (pain reduction > 75% according to visual analog scale for at least 12 months) following surgery and in the 3-year follow-up period.

**Discussion:**

Conservative treatment is first-choice treatment for managing PS. Surgery was indicated when the patient did not achieve satisfactory pain relief with conservative treatment. Surgical resection of the piriformis muscle has been shown to be effective and feasible.

**Conclusion:**

Piriformis resection is effective and can be a feasible option for the treatment of PS following failure of appropriate conservative treatment(s).

## Highlights

• Some of piriformis syndrome intractable with conservative treatment.
• Surgery was indicated for intractable piriformis syndrome.
• Piriformis resection can be a feasible option for intractable piriformis syndrome.
• Piriformis resection decrease visual analog scale in intractable piriformis syndrome.

## 1 Introduction

Piriformis syndrome (PS) refers to pain caused by impingement of the sciatic nerve by the piriformis muscle, resulting in irritation of the sciatic nerve and the generation of symptoms including low back, hip, buttock, and leg pain [1]. The prevalence of PS in individuals who experience chronic low back and sciatic pain is approximately 0.5%–17% [2].

PS is frequently unrecognized and/or misdiagnosed by physicians as spinal problems or other inflammatory conditions due to the lack of a gold standard tool for definitive diagnosis [3]. Other modalities can be used to exclude other pathologies, including electromyography (EMG), computed tomography (CT), and magnetic resonance neurography (MRN). Furthermore, steroid injection of the piriformis muscle, with or without neurolysis, can be performed to confirm PS [4].

Conservative treatment is the first choice in treating patients with PS. Activity modification, anti-inflammatory drugs, physical therapy, injection of anesthetics, steroids, or neurotoxins may alleviate symptoms. However, in some cases, conservative treatment results in only minor symptom relief. Surgical decompression can be considered as a last resort in treating PS. After failed response to conservative treatment, > 80% of patients (of 62 cases) were reported to experience significant pain relief immediately after surgical resection of the piriformis, with most experiencing long-term relief [1,5,6]. Here, we report our experience and discuss the surgical methods for managing PS. This work has been reported in line with the SCARE guideline [7]. This work has been registered at http://www.researchregistry.com (researchregistry6134).

## 2 Presentation of case

A 72-year-old man visited the authors’ hospital outpatient clinic complaining of a 2-year history of left buttock pain radiating to his left leg. He also experienced a tingling sensation, and diffuse or poorly localized radiating pain in the left leg. These symptoms were aggravated by the activities of daily living, especially sitting. Patient frequently sit at hard wood chair. On radiological examination, dynamic lumbosacral spinal X-ray and magnetic resonance imaging revealed that the spine was stable, with facet hypertrophy and mild central canal stenosis at the level of lumbar 4–5.

On physical examination, the left foot was externally rotated in the resting position. The straight leg raise test was positive at 40° and the Flexion Adduction Internal Rotation (i.e., FAIR) test was confirmed after performing provocation maneuvers. Piriformis muscle stretch pain and tenderness on palpation were noted. The patient’s response to buttock pain, assessed according to a visual analog scale (VAS) scored 0–10, was 8. No sensorineural deficits were present over the lower extremity. Based on the patient’s history and physical examinations, a provisional primary diagnosis of myofascial pain syndrome of the piriformis muscle was made.

To manage the patient’s condition and confirm the diagnosis of PS, injection blocking of the piriformis muscle using steroid was performed. The symptoms were significantly reduced (VAS score, 3) following the procedure, and he was able to perform his daily activities by himself. During follow-up, however, the symptoms recurred 3 months later (VAS score, 7). A second piriformis injection was performed with the same result. After failed conservative treatment and a confirmed diagnosis of PS, we performed surgical resection of the piriformis muscle as the last resort.

Intraoperative findings revealed pinching of the left sciatic nerve by the piriformis muscle. After the sciatic nerve was released by resecting the piriformis muscle, steroids were administered to the area surrounding the nerve and tissue to address inflammation along with adhesion barrier gel to prevent adhesion after surgery.

### 2.1 Surgical approach

**Fig. 1:**
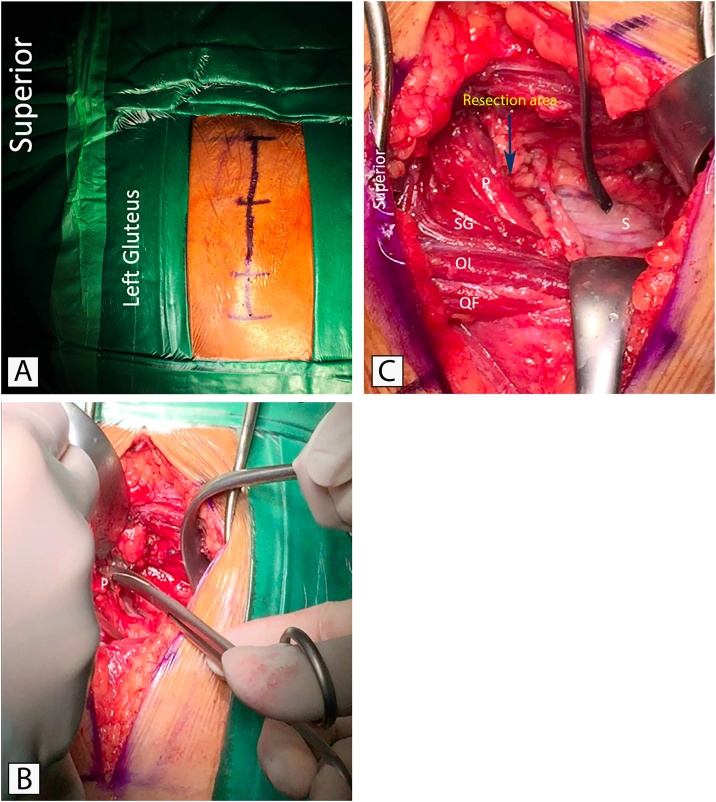
Intraoperative photographs. A. Surgical incision planning. B. The inner side of the piriformis muscle (P) was resected. C. Dissected fibrous tissues around the sciatic nerve, SG: superior gemellus muscle, OI: obturator internus muscle, QF: quadratus femoris muscle.

Surgery was performed by the author, with the patient in the prone position. A linear skin incision was made > 10 cm over the greater trochanter. The piriformis muscle was located at the posterior aspect of the greater trochanter and obturator internus muscle (Fig. 1A). The gluteus maximus was bluntly dissected in the direction of its fibers by blunt dissection until the piriformis muscle was exposed. The sciatic nerve was explored and found below the piriformis muscle, with inflamed tissue surrounding the sciatic nerve. Decompression was performed by partial resection of the inner part of the piriformis muscle that compressed the sciatic nerve (Fig. 1B) [6,8]. Subsequently, the fibrous tissues around the sciatic nerve were carefully dissected to avoid damaging the nerve (Fig. 1C). Triamcinolone acetonide (20 mg) (Flamicort, Dexa Medica, Palembang, Indonesia), a synthetic corticosteroid, and barrier adhesion gel (Mediclore, Jakarta, Daewoong Pharmaceutical Company, Indonesia), were applied to the sciatic nerve and its surrounding tissues to achieve an anti-inflammatory effect and prevent adhesion.

### 2.2 Result

The surgical outcome was considered to be satisfactory if there was a significant decrease in pain response according to VAS, with > 50% reduction during 1-year follow-up after surgery. The patient exhibited satisfactory results that lasted > 3 years after surgery, with no neurological deficits or surgical complications. The symptoms were significantly relieved (VAS score, 2), with no neurological deficits or complications after surgery. During the 3 years of follow-up, the patient experienced satisfactory pain relief, patient evaluated in every 3 months after surgery (VAS score, 2; pain reduction > 75% according to VAS for at least 12 months) following surgery and in the 3-year follow-up period (yearly evaluated).

## 3 Discussion

**Fig. 2:**
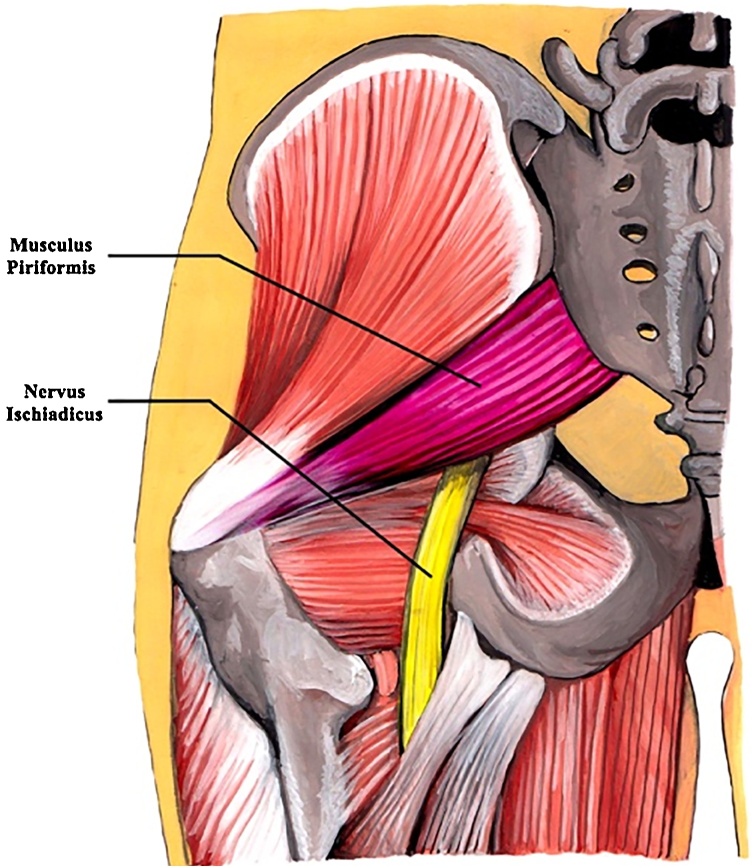
Schematic location of the sciatic nerve travelling below the piriformis muscle.

PS is a neuromuscular condition that affects between 5% and 36% of patients with chronic low back and sciatic pain. PS is believed to be more common in women than men, mainly occurring in the fourth to fifth decades of life [8,9]. The sciatic nerve is formed by L4-S3 nerve roots and commonly passes anteriorly to the piriformis muscle (Fig. 2). However, in 17% of cases, the sciatic nerve and its branches pass through the piriformis muscle, as observed in our patient intraoperatively, thus causing a selective or dermatomal type of pain when the nerve or its branch is compressed [10]. Typical presentation of piriformis pain would be from the lower back or buttock to the knee, as exhibited by our patient. PS is mostly caused by microtrauma to the buttocks, leading to inflammation of soft tissue, muscle spasm, or both, with resulting nerve compression [11]. Microtrauma may result from the overuse of piriformis muscle, long-time repetitive sitting on a hard-surface chair, and by direct compression, such as in “wallet neuritis” [12].

Clinical assessment of patients with PS is difficult because the symptoms are similar to and may be biased by other lumbar or intra- or extra-articular hip diseases. Physical examination tests have been used for the clinical diagnosis of sciatic nerve entrapment including the Lasègue, Pace, Freiberg, Beatty, FAIR, and piriformis stretch tests. Fishman’s clinical criteria can also be used to assess PS (Table 1) [1,13]. A positive FAIR test and Lasegue’s sign were observed in our patient, indicating that the pain originated from the gluteal or sciatic area, mostly due to compression of the sciatic nerve by the piriformis muscle [13].

**Table 1:** Fishman’s clinical criteria for piriformis syndrome.

1. Positive Laseque sign at 45°
2. Tenderness at the sciatic notch
3. Increased pain in the sciatic distribution with the thigh in the FAIR (Flexion Adduction Internal Rotation) position
4. Electrodiagnostic studies that exclude myopathy or neuropathy

Several modalities can be used to assess PS, including EMG, CT, and MRN. However, there is no gold standard diagnostic tool for PS. These modalities can be used to help exclude other pathologies that may elicit symptoms [4]. Aside from clinical history and physical examinations, steroidal blocking injection, with or without neurolysis, of the piriformis muscle remains a reliable method of confirming a diagnosis of PS. In a study by Filler et al., patients with PS demonstrated a good response to local anesthetic and steroid injections of the piriformis muscle [14]. In our case, the patient also responded to local steroid injection, but lasted for only 3 months after every injection.

Conservative treatment is first-choice treatment for managing PS. Conservative treatment for patients with PS includes activity modification (education about changing habitual postures or physical activities), anti-inflammatory drugs, physical therapy, injection of local anesthetics or corticosteroids, and botulinum neurotoxin injections [1,5,8,15]. Our patient also underwent conservative treatment before surgery. At the beginning of the disease course, the patient demonstrated good response to medications and physical therapies, lasting for 3 years. However, as the condition worsened, he failed to respond to conservative treatment(s).

Surgery was indicated when the patient did not achieve satisfactory pain relief with conservative treatment. The goal of surgery is to release the sciatic nerve by resecting the piriformis muscle that compresses the nerve. Surgical resection of the piriformis muscle has been shown to be effective and feasible [6,8]. In a study by Han et al. in 2017, conservative treatment failed in 12 of 239 patients, who subsequently underwent surgical procedures. After surgical resection of the piriformis, 10 (83%) patients experienced satisfactory results based on VAS scores, which were recorded before and after surgery. No postoperative complications were observed during the 1-year follow-up [8]. A similar result was reported by Filler et al., in which 62 (80%) surgical patients experienced significant pain relief with no surgical complications [14]. In our case, we performed surgical resection of the piriformis using the method described by Kobbe et al. [6]. Steroid and anti-adhesion gel were applied to achieve anti-inflammatory effects and to prevent adhesion.

## 4 Conclusion

The diagnosis of PS can be vague and often elusive due to its clinical presentation, which mimics other pathologies of sciatic pain. Consideration of patient history, clinical presentation, and response to diagnostic or therapeutic steroid or anesthetic local injection of the piriformis muscle are essential to confirm the diagnosis of PS and provide proper treatment. The surgical procedure for resecting the piriformis muscle is not complicated and yields a satisfactory result. Surgical treatment can be a last-resort option to treat refractory PS that fails conservative treatment.

## Declaration of Competing Interest

None.

## Sources of funding

None.

## Ethical approval

All of the procedures performed in this study involving human participants were in accordance with the ethical standards of the institutional research committee.

## Consent

All of the patient had sign informed consent for the surgery.

Sorry, we could not got written informed consent from the patient, because we did not saw the patient again in last one year and we could not contact the patient, patient identity doesn’t seen in this case report and we have permission from head of neurosurgery Department in our Hospital

## Author contribution

Achmad Fahmi, MD, PhD: study concept or design, data collection, data analysis or interpretation, writing the paper.

Mustaqim Apriyansa Rahmadhan, MD.: study concept or design, writing paper.

Dirga Rachmad Aprianto, MD: study concept or design.

Heri Subianto, MD: study concept and critical revised article.

Agus Turchan, MD, PhD: study concept, critical revised article and supervising.

## Registration of research studies

1. Name of the registry: http://www.researchregistry.com
2. Unique identifying number or registration ID: researchregistry6134
3. Hyperlink to your specific registration (must be publicly accessible and will be checked):

## Guarantor

Achmad Fahmi, MD, PhD

Department of Neurosurgery, Faculty of Medicine, Universitas Airlangga, Indonesia

Agus Turchan, MD, PhD

Head of Neurosurgery Department, Faculty of Medicine, Universitas Airlangga, Indonesia

## Provenance and peer review

Not commissioned, externally peer-reviewed.
